# Multimorbidity Among Migrant and Non-Migrant Ghanaians: The RODAM Study

**DOI:** 10.3389/ijph.2021.1604056

**Published:** 2021-12-31

**Authors:** Anna Marzà-Florensa, Daniel Boateng, Charles Agyemang, Erik Beune, Karlijn A. C. Meeks, Silver Bahendeka, Naomi Levitt, Kerstin Klipstein-Grobusch

**Affiliations:** ^1^ Julius Global Health, Julius Center for Health Sciences and Primary Care, University Medical Center Utrecht, Utrecht University, Utrecht, Netherlands; ^2^ Department of Public and Occupational Health, Amsterdam Public Health Research Institute, Amsterdam University Medical Centers, University of Amsterdam, Amsterdam, Netherlands; ^3^ Center for Research on Genomics and Global Health, National Human Genome Research Institute, National Institutes of Health, Bethesda, MD, United States; ^4^ Department of Internal Medicine, Mother Kevin Post Graduate Medical School, Uganda Martyrs University, Nkozi, Uganda; ^5^ Division of Endocrinology, Department of Medicine, University of Cape Town, Cape Town, South Africa; ^6^ Department of Epidemiology and Biostatistics, School of Public Health, University of the Witwatersrand, Johannesburg, South Africa

**Keywords:** multimorbidity, low- and middle-income countries, epidemiology, global health, non-communicable diseases, migration, urbanization

## Abstract

**Objectives:** Multimorbidity is a growing public health concern due to the increasing burden of non-communicable diseases, yet information about multimorbidity in low- and middle-income countries and migrant populations is scarce. We aimed to investigate the distribution and patterns of multimorbidity in rural and urban areas in Ghana and Ghanaian migrants in Europe.

**Methods:** The RODAM cross-sectional study included 4,833 participants. Multimorbidity was defined as presence of multiple non-communicable chronic conditions. Patterns were determined from frequent combination of conditions. Prevalence ratios were estimated by logistic regression.

**Results:** Prevalence of multimorbidity was higher in women and in urban Ghana and Europe. We observed a cardiometabolic pattern in all sites as well as circulatory-musculoskeletal and metabolic-musculoskeletal combinations in Ghana. Multimorbidity prevalence ratios were higher in Europe (men 1.47, 95% CI 1.34–1.59, women 1.18, 1.10–1.26) and urban Ghana (men 1.46, 1.31–1.59, women 1.27, 1.19–1.34).

**Conclusion:** Distribution and patterns of multimorbidity differed by sex and site. With a higher burden of multimorbidity in urban areas, prevention strategies should focus on forestalling its increase in rapidly growing rural areas.

## Introduction

Multimorbidity is defined as the presence of multiple chronic conditions [1]. Multimorbidity is an increasingly important public health concern due to ageing populations and the subsequent growing burden of non-communicable diseases (NCDs). Multimorbidity results in more complex care and management of patients, lower quality of life and higher mortality rates [1]. This is particularly important in low- and middle-income countries (LMICs), since many LMICs are undergoing a health and demographic transition resulting in increased burden of NCDs [2, 3]. With NCDs becoming more common, more people in LMICs are likely to suffer from multiple chronic conditions.

Previous research reported prevalence estimates of multimorbidity ranging from 3.2 to 90.5% in LMICs [4, 5], generally showing prevalence estimates comparable to those from high-income countries (HICs) [5, 6]. Commonly reported risk factors for multimorbidity include older age, female sex, low socioeconomic status, and, in the case of international migrants, longer period of stay in the host country [5, 7–12]. In addition, several studies have identified combinations of chronic conditions that often present together (i.e., specific patterns of multimorbidity), including clustering of cardiometabolic, cardiovascular and respiratory, cardiovascular and articular, and mental and articular conditions [5, 9, 10, 13–15]. Although studies on multimorbidity patterns in migrants are scarce, Diaz et al. [8] described that these were similar between Norwegian-born population and migrants in Norway, with some migrant groups presenting more pattern complexity.

Migration often entails changes in social and environmental exposures, usage of healthcare systems and raised prevalence of chronic conditions. These migration-specific factors may increase the risk of presenting multiple chronic conditions and the adverse health outcomes associated with it. Studying migration and multimorbidity is a research priority [1, 16], yet literature on this topic is scarce. To our knowledge, this is the first study to assess the distribution and patterns of multimorbidity in migrants to HICs compared to their population of origin.

Understanding the clustering of chronic conditions in similar populations in different settings will contribute to broaden the knowledge in the fields of multimorbidity and migrant health, eventually facilitating efficient management of patients presenting multiple chronic conditions.

The Research on Obesity and Diabetes among African Migrants (RODAM) Study investigates NCDs in Ghanaian born residents in Ghana and their counterparts that have migrated to Europe. Previous analyses in the RODAM Study show differences in the burden of type 2 diabetes, hypertension and cardiovascular disease (CVD) risk between sub-Saharan African migrant populations residing in different European countries and their home country compatriots [17–19]. In this paper, we explored the distribution of multimorbidity and the most frequent combinations of chronic conditions among Ghanaian residents in rural and urban areas in Ghana and migrants of Ghanaian origin in Europe.

## Methods

### Study Design and Community Involvement

The RODAM Study is a cross-sectional multi-centre study carried out between 2012 and 2015. The rationale and framework of the study, as well as the recruitment of the study population, are described in detail elsewhere [20]. In short, the study included Ghanaian adults living in rural and urban areas in Ghana, and urban areas in three European locations: Amsterdam, London and Berlin. In Ghana, participants were randomly selected from a list of 30 enumeration areas in the Ashanti region. In Amsterdam, participants were randomly selected from the municipality’s registry. In London and Berlin, potential participants were identified from lists of Ghanaian organizations, from which individuals aged 25 years or older were invited to participate [20].

Community leaders were involved from early stages in the project and their endorsement was key throughout the study. They were identified through community organizations such as churches or mosques in all study sites. A community support group including community leaders and media groups was set up to discuss the protocol and recruitment strategies, and ensure these were acceptable to the community. Community leaders collaborated in setting up the research, supported a pilot for the use of validated questionnaires and relevant questions, and actively collaborated in recruitment of study subjects. The community actively contributed to the planning and dissemination of study results, which was carried out through community media outlets and organizations [20].

### Study Population

Overall, 6,385 individuals agreed and participated in Ghana, London, Berlin and Amsterdam [20]. Participants who did not complete the physical examination or did not provide blood samples were excluded. For this analysis, we included participants aged 25–70 years, since not all study sites included individuals outside this age range. We excluded participants without complete data on multimorbidity (826 participants). A total of 4,833 participants were available for data analysis: 2,526 in Europe, 1,377 in urban Ghana and 990 in rural Ghana (Supplementary Figure S1).

The study was conducted according to the guidelines contained in the Declaration of Helsinki. All procedures were approved before data collection by the respective ethics committees in Ghana, the Netherlands, the United Kingdom and Germany [20]. Written informed consent from each participant was obtained prior to enrolment.

### Data Collection

Data collection was conducted using questionnaires, physical examinations and laboratory measures from blood and urine samples. Data collection approaches were well-standardized: standard operating procedures and standardized tools were used in all sites, and staff received specific training. Questionnaires were used to collect information on independent variables related to demographics, socio-demographics, lifestyle and diseases status. In our analysis, we included demographic variables (study site and age), sociodemographic variables (employment status and educational level), health-related behaviour variables (current smoking status, physical activity and alcohol consumption), and variables on disease status.

Physical examinations were performed to measure anthropometric variables and blood pressure. Samples of fasting venous blood and urine were collected, frozen and subsequently shipped and analysed centrally at Charité Universitätsmedizin Berlin, Germany.

### Variable Definition

Employment status was categorized into three groups: employed, unemployed (including unemployed and looking for work and students), and other (including participants unable to work, on social benefits, full-time homemakers and retired). Educational level was grouped into three categories according to the highest educational level attained: none-low (never attended school, elementary school, lower vocational or secondary schooling), intermediate (intermediate vocational, secondary or higher secondary schooling), and high (higher vocational schooling or university).

Current smoking status was classified into three categories: current, never or former smoker. Physical activity was defined as low, moderate or high according to the International Physical Activity Questionnaire scoring protocol (IPAQ) [21]. Alcohol consumption was classified in two categories according to the European Society of Cardiology Guidelines [22]: moderate or no consumption and excessive consumption.

The aim of the RODAM Study is to investigate risk factors for obesity and diabetes. Information on other conditions was also collected, allowing for assessment of multimorbidity in the RODAM primary dataset. Self-reported chronic conditions with an overall prevalence lower than 2% (including lung disease and cancer) were excluded. Blood pressure (BP) was measured three times in a sitting position after at least 5 min of rest and hypertension was defined as systolic BP ≥ 140 mm Hg, or diastolic BP ≥ 90 mmHg, or being on antihypertensive medication [18]. Obesity was defined as body mass index (BMI) ≥ 30 kg/m^2^. Type 2 diabetes mellitus was defined according to the World Health Organization criteria: fasting plasma glucose ≥7.0 mmol/L, current use of medication prescribed for diabetes, or self-reported diabetes [23]. Hypercholesterolemia was defined as total cholesterol level ≥5 mmol/L. Estimated glomerular filtration rate and albuminuria were used to estimate severity of chronic kidney disease according to the 2012 Kidney Disease: Improving Global Outcomes (KDIGO) classification [24]: low, moderately increased, high and very high risk. Participants with moderately increased, high and very high risk of chronic kidney disease considered to present chronic kidney disease [25]. Current CVD (intermittent claudication, angina pectoris and possible myocardial infarction) was determined with the Rose Questionnaire [26]. Rheumatic disorders were assessed *via* questionnaires and included inflammatory rheumatism, chronic rheumatism or rheumatoid arthritis diagnosed by a doctor. Depressive symptoms were defined with a score of ≥10 on the Patient Health Questionnaire (PHQ-9) [27]. This questionnaire assesses if the patient has experienced these situations in the previous 2 weeks: feeling down or little pleasure in doing things, appetite and sleeping difficulties, tiredness, concentration problems, feelings of failure and self-harm, feeling restless or moving very slowly. The questionnaire in the RODAM Study further included migration-related items such as financial demands from the family in the country of origin, homesickness and difficulties adapting to the host country. Further details on the definition of study variables are provided in Supplementary Table S1.

### Outcome Definition

Multimorbidity was defined as having two or more of the following chronic non-communicable conditions: hypertension, obesity, type 2 diabetes mellitus, hypercholesterolemia, CVD, chronic kidney disease, rheumatic disorders, or depressive symptoms. Multimorbidity was expressed as a dichotomous outcome: participants with two or more chronic conditions were considered to present multimorbidity. Additionally, we categorized the number of conditions to explore the distribution of multimorbidity in more detail: participants were grouped into absence of multimorbidity (i.e., having none or one condition), having two or three conditions, or presenting with four or more conditions Multimorbidity patterns were defined as combinations of two or more conditions with a prevalence of at least 5% and named according to the International Classification of Disease 11 (ICD-11) disease groups [28].

### Statistical Analysis

The study population characteristics were expressed as number and percentage of participants for categorical variables and means with standard deviation (SD) for continuous variables. The prevalence of individual conditions and multimorbidity was reported as percentage of participants affected per study site. Age-standardized prevalence rates of diseases and multimorbidity were calculated using the direct method [29] with the total RODAM population as the standard population.

The percentage of participants with the four most common conditions alone or combinations of two, three or four conditions was reported and displayed in Euler diagrams per study site.

The association between geographical site on risk of multimorbidity was assessed with logistic regression analysis. Three models were built with rural Ghana as the reference study site. We used a stepwise process to estimate the effect of study site on multimorbidity while adjusting for variables that may influence the association. Model one included only study site and age; model two consisted of model one plus education level and employment status; lastly, model three included model two plus current smoking status. The results of the logistic regression were reported as prevalence ratios (PR) and 95% confidence intervals (CI). Available case analysis was used to deal with missing data. Sensitivity analysis performed after multiple imputation showed similar results to the available case analysis. All analyses were stratified by sex. RStudio version 1.0.456 [30] was used for statistical analysis and a two-sided *p*-value of <0.05 was considered statistically significant.

## Results

The characteristics of the study population are shown in Table 1. A total of 1,828 men and 3,005 women were included in the present analysis. Mean (SD) age was 46.8 (11.2) years and 45.6 (10.6) years in men and women respectively. The proportion of participants achieving a high level of education was lowest in women in rural Ghana and highest in men in Europe (21.5%), while employment was lowest in Europe and highest in rural Ghana (90.2% of women).

**TABLE 1:** Baseline characteristics of the study population.

	Men				Women			
	Europe (*n* = 1075)	Urban Ghana (*n* = 386)	Rural Ghana (*n* = 367)	Total (*n* = 1828)	Europe (*n* = 1451)	Urban Ghana (*n* = 991)	Rural Ghana (*n* = 563)	Total (*n* = 3005)
Age (Years): Mean, SD	47.2 (10.4)	46.5 (11.8)	46.2 (12.8)	46.8 (11.2)	45.8 (9.4)	44.7 (11.2)	46.5 (12.5)	45.6 (10.6)
Curernt smoking status								
Current smoker	86 (8.0)	13 (3.4)	22 (6.0)	121 (6.6)	24 (1.7)	1 (0.1)	0 (0)	25 (0.8)
Never smoked	851 (79.2)	311 (80.6)	284 (77.4)	1446 (79.1)	1353 (93.2)	968 (97.7)	555 (98.6)	2876 (95.7)
Ex-Smoker	137 (12.7)	62 (16.1)	61 (16.6)	260 (14.2)	68 (4.7)	21 (2.1)	5 (0.9)	94 (3.1)
Missing	1 (0.1)	0 (0)	0 (0)	1 (0.1)	6 (0.4)	1 (0.1)	3 (0.5)	10 (0.3)
Alcohol Consumption								
No alcohol/Not excessive	1001 (93.1)	377 (97.7)	341 (92.9)	1719 (94.0)	1403 (96.7)	987 (99.6)	560 (99.5)	2950 (98.2)
Excessive	74 (6.9)	9 (2.3)	26 (7.1)	109 (6.0)	47 (3.2)	4 (0.4)	3 (0.5)	54 (1.8)
Missing	0 (0)	0 (0)	0 (0)	0 (0)	1 (0.1)	0 (0)	0 (0)	1 (0.0)
Physical Activity								
Low	243 (22.6)	85 (22.0)	39 (10.6)	367 (20.1)	332 (22.9)	398 (40.2)	133 (23.6)	863 (28.7)
Moderate	170 (15.8)	71 (18.4)	63 (17.2)	304 (16.6)	288 (19.8)	155 (15.6)	130 (23.1)	573 (19.1)
High	455 (42.3)	224 (58.0)	262 (71.4)	941 (51.5)	540 (37.2)	432 (43.6)	299 (53.1)	1271 (42.3)
Missing	207 (19.3)	6 (1.6)	3 (0.8)	216 (11.8)	291 (20.1)	6 (0.6)	1 (0.2)	298 (9.9)
Educational level								
None-Low	574 (53.4)	263 (68.1)	292 (79.6)	1129 (61.8)	923 (63.6)	877 (88.5)	533 (94.7)	2333 (77.6)
Intermediate	259 (24.1)	85 (22.0)	52 (14.2)	396 (21.7)	341 (23.5)	87 (8.8)	18 (3.2)	446 (14.8)
High	231 (21.5)	37 (9.6)	23 (6.3)	291 (15.9)	170 (11.7)	27 (2.7)	11 (2.0)	208 (6.9)
Missing	11 (1.0)	1 (0.3)	0 (0)	12 (0.7)	17 (1.2)	0 (0)	1 (0.2)	18 (0.6)
Employment status								
Employed	804 (74.8)	332 (86.0)	327 (89.1)	1463 (80.0)	906 (62.4)	843 (85.1)	508 (90.2)	2257 (75.1)
Unemployed^a^	132 (12.3)	16 (4.1)	13 (3.5)	161 (8.8)	148 (10.2)	42 (4.2)	20 (3.6)	210 (7.0)
Other^b^	130 (12.1)	38 (9.8)	27 (7.4)	195 (10.7)	376 (25.9)	106 (10.7)	35 (6.2)	517 (17.2)
Missing	9 (0.8)	0 (0)	0 (0)	9 (0.5)	21 (1.4)	0 (0)	0 (0)	21 (0.7)
Multimorbidity								
Yes	534 (49.7)	195 (50.4)	114 (31.0)	843 (46.1)	887 (61.1)	642 (64.8)	280 (49.8)	1809 (60.2)
No	541 (50.3)	191 (49.6)	254 (69.0)	985 (53.9)	564 (38.9)	349 (35.2)	283 (50.2)	1196 (39.8)

a Includes unemployed and students.

b Includes unable to work, on social benefits, full-time homemakers and retired.

The values represent mean (standard deviation) for continuous variables, and number of participants (percentage) for categorical variables. Research on Obesity and Diabetes among African Migrants (RODAM) Study. Ghana, United Kingdom, Germany, The Netherlands 2012–2015.

Current smoking and excessive alcohol consumption were more common in men than in women, and in Europe compared to Ghana.

**FIGURE 1:**
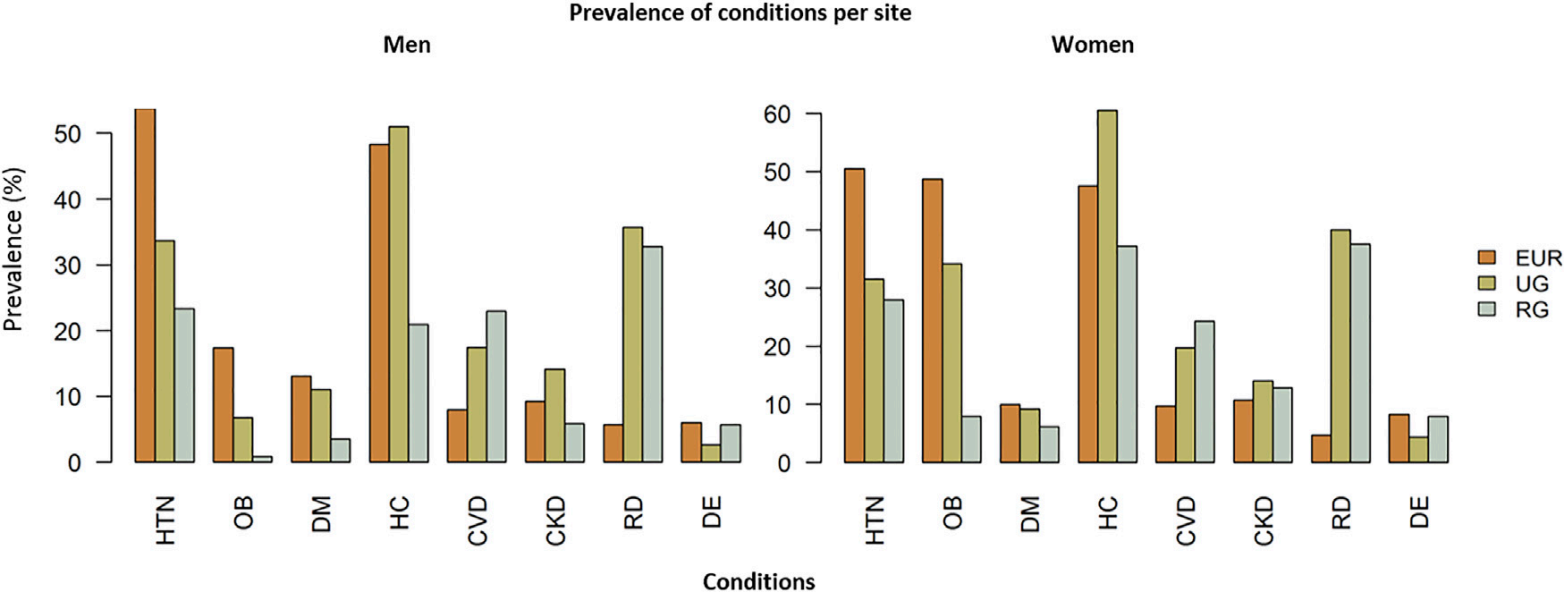
Age-adjusted prevalence of individual chronic conditions per site in men **(A)** and women **(B)**. Abbreviations: EUR, Europe; UG, urban Ghana; RG, rural Ghana; HTN, hypertension; OB, obesity; DM, diabetes mellitus; HC, hypercholesterolemia; CVD, cardiovascular disease; CKD, chronic kidney disease; RD, rheumatic disorders; DE, depressive symptoms. Research on Obesity and Diabetes among African Migrants (RODAM) Study. Ghana, United Kingdom, Germany, Netherlands 2012–2015.

The age-standardized prevalence of the individual chronic conditions by sex and study site is displayed in Figure 1, and by sex, study site and age group in Supplementary Table S2. The prevalence of hypertension, obesity and diabetes was lowest in rural Ghana and highest in Europe. The proportion of participants with hypercholesterolemia ranged from 20.9% of men in rural Ghana to 60.5% of women in urban Ghana. Prevalence of pre-existing CVD was lowest in Europe and highest in rural Ghana. Chronic kidney disease was observed in 14.0% women in urban Ghana and 10.8% of women in Europe. The proportion of participants with rheumatic disorders was lowest in Europe and highest in urban Ghana: 4.7% of women in Europe and 40.0% of women in urban Ghana had rheumatic disorders and 6.0% of European men and 39.4% of men in urban Ghana presented rheumatic disorders. The prevalence of depressive symptoms was highest in Europe and lowest in urban Ghana.

### Prevalence and Patterns of Multimorbidity

**FIGURE 2:**
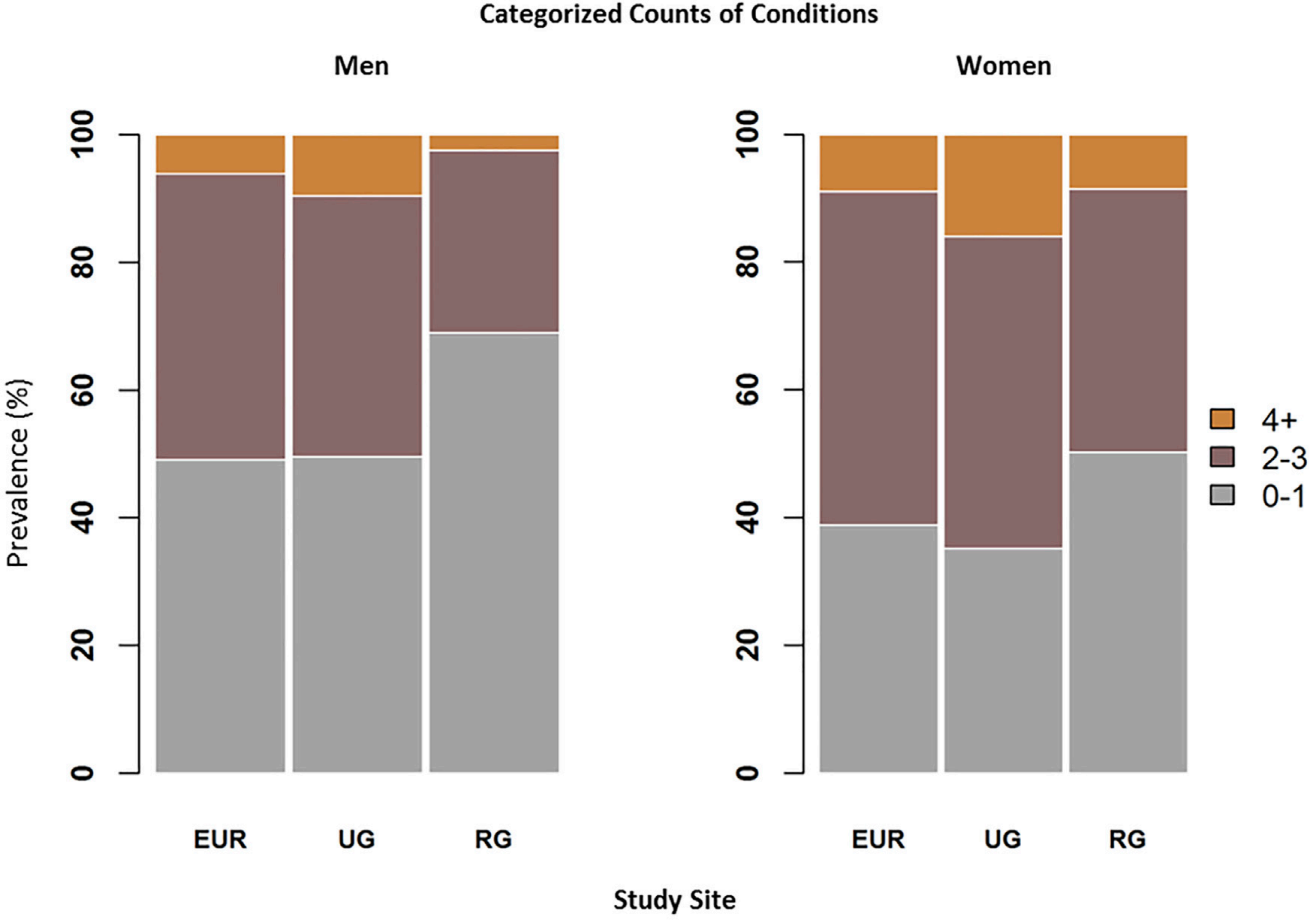
Age-adjusted prevalence of categorized chronic conditions counts in men **(A)** and women **(B)**: 0–1 conditions (no multimorbidity), 2–3 conditions, and 4 or more conditions. Abbreviations: EUR, Europe; UG, urban Ghana; RG, rural Ghana. Research on Obesity and Diabetes among African Migrants (RODAM) Study. Ghana, United Kingdom, Germany, Netherlands 2012–2015.

The prevalence of multimorbidity was, in men and women respectively, 51.0% (95% CI 48.0%–54.0%) and 61.1% (95% CI 58.6%–63.6%) in Europe; 50.5% (95% CI 45.5%–55.5%) and 64.8% (95% CI 61.8%–67.8%) in urban Ghana; and 31.1% (95% CI 26.3%–35.8%) and 49.7% (95% CI 45.6%–53.9%) in rural Ghana. Multimorbidity was more prevalent in women than in men (Figure 2) and in participants of older age (Supplementary Table S2).

For both men and women, rural Ghana had the lowest prevalence of multimorbidity: 28.6% (95% CI 24.0%–33.2%) of men and 41.2% (95% CI 37.1%–45.3%) of women presented with 2 or 3 conditions, and only 2.5% (95% CI 0.9%–4.0%) of men and 8.6% (95% CI 6.2%–10.8%) of women presented with 4 or more conditions. Prevalence estimates of multimorbidity in Europe and urban Ghana were very similar. The highest prevalence of multimorbidity was observed in urban Ghana: 40.8% (95% CI 36.0%–45.8%) of men and 48.9% (95% CI 45.7%–52%) of women had 2 or 3 conditions and 9.6% (95% CI 6.6%–12.5%) of men and 16.0% (95% CI 13.7%–18.2%) of women presented 4 or more conditions.

**FIGURE 3:**
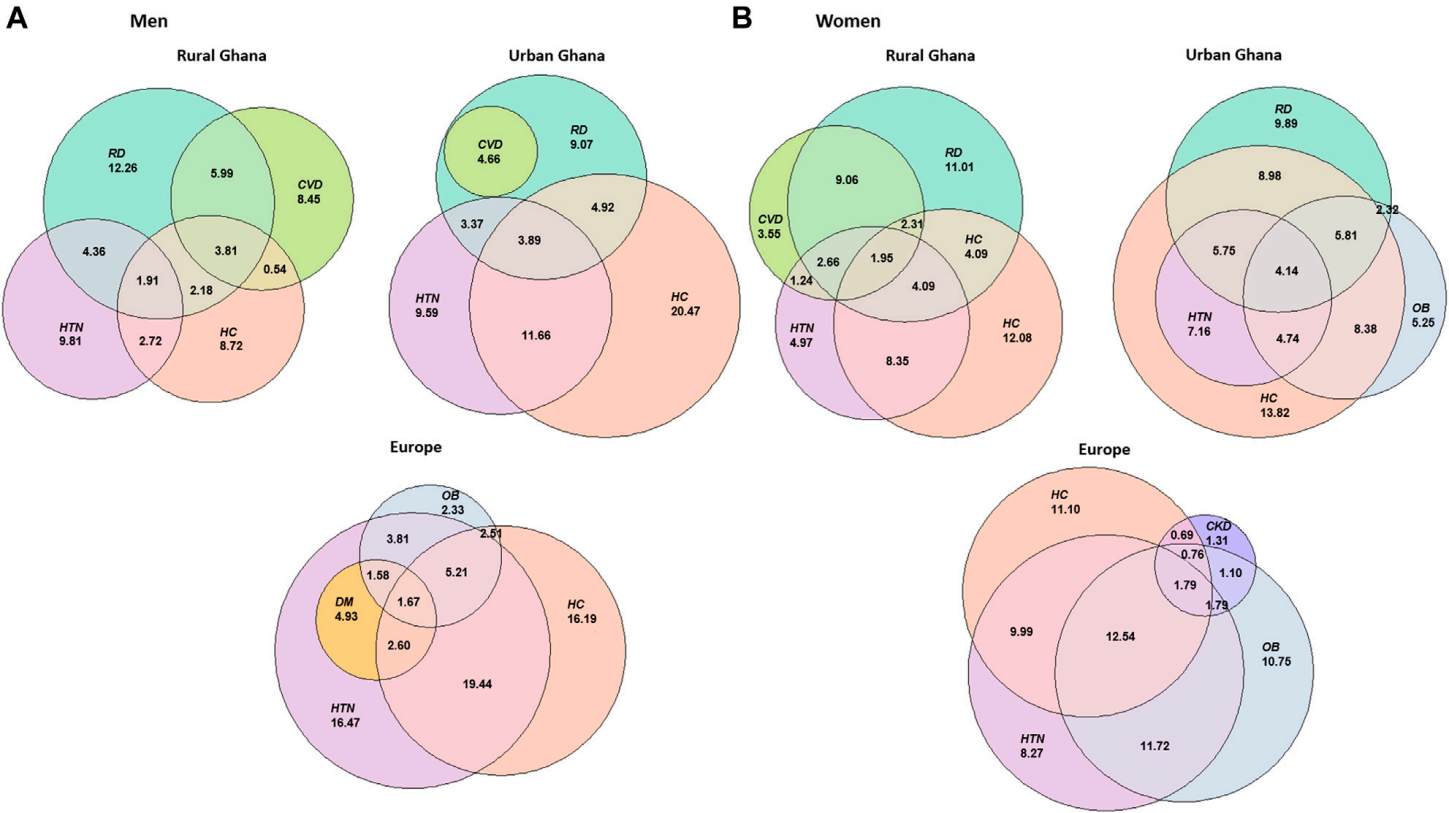
Euler diagrams displaying the percentage of participants with each chronic condition or combinations of two, three or four chronic conditions in Europe, urban Ghana and rural Ghana in men **(A)** and women **(B)**. Colours represent different health conditions, abbreviated as: HTN, hypertension; OB, obesity; DM, diabetes mellitus; HC, hypercholesterolemia; CVD, cardiovascular disease; CKD, chronic kidney disease; RD, rheumatic disorders; DE, depressive symptoms. Research on Obesity and Diabetes among African Migrants (RODAM) Study. Ghana, United Kingdom, Germany, Netherlands 2012–2015.

Figure 3 displays the percentage of participants with prevalent combinations of chronic conditions in each study site in men (Figure 3A) and women (Figure 3B). Co-existing cardio-metabolic morbidities were present in all groups except men in rural Ghana and were more common in Europe: 11.7% (10.0%–13.4%) of Ghanaian women in Europe had obesity and hypertension, and 12.5% (10.8%–14.2%) had hypercholesterolemia as well.

Rheumatic disorders were often presented in combination with other conditions in Ghana. For instance, 9.1% (95% CI 6.7%–11.5%) of women and 6.0% (95% CI 3.6%–8.4%) of men in rural Ghana presented CVD and rheumatic disorders (dominance of circulatory-musculoskeletal morbidities). A metabolic-musculoskeletal combination was observed in women in urban Ghana, with 9.0% (95% CI 7.2%–10.8%) having hypercholesterolemia and rheumatic disorders, and 5.9% (95% CI 4.4%–7.4%) having hypercholesterolemia, obesity and rheumatic disorders. 5.8% (95% CI 4.4%–7.3%) of women in urban Ghana presented a cardiometabolic-musculoskeletal co-existence, including hypercholesterolemia, hypertension and rheumatic disorders. It was also in women in urban Ghana that a metabolic pattern was observed, with a prevalence of obesity and hypercholesterolemia of 8.4% (95% CI 6.7%–10.1%).

**FIGURE 4:**
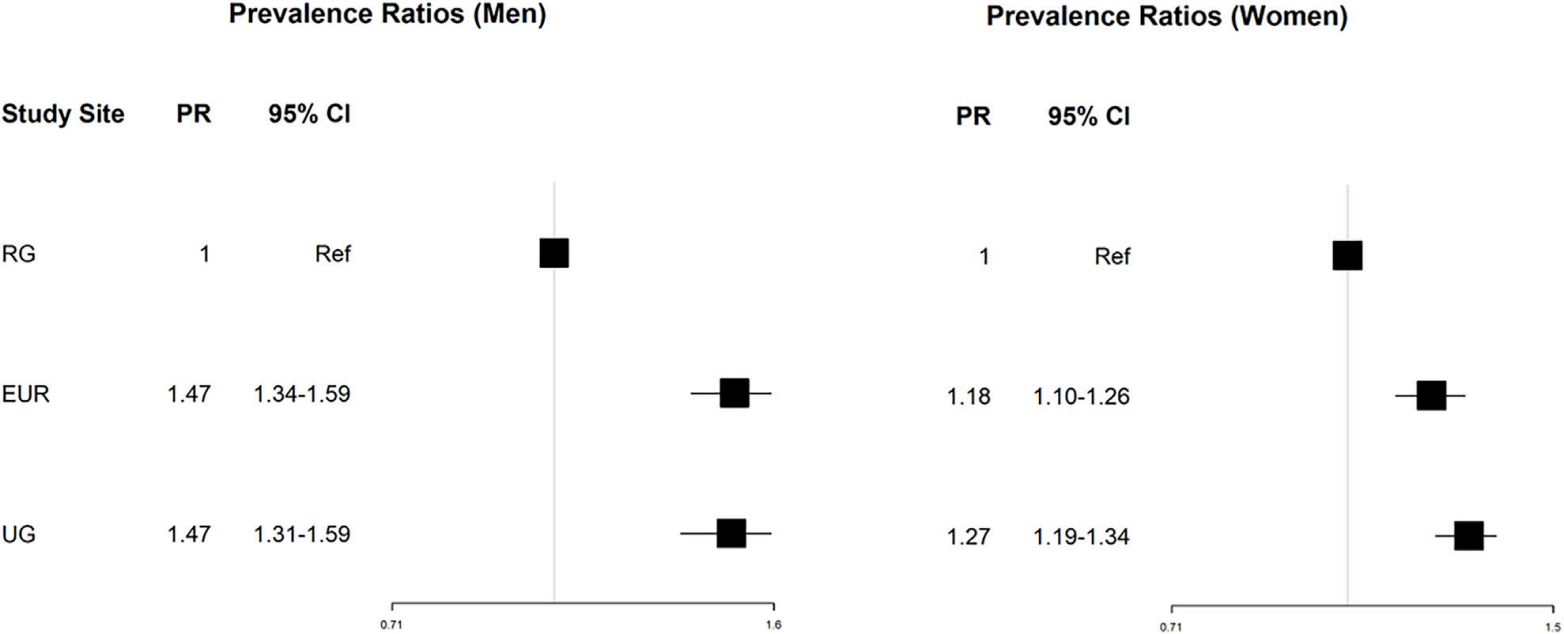
Forest plot displaying the prevalence ratios, and 95 confidence intervals of MM in the study locations compared to the reference (rural Ghana). Abbreviations: RG, rural Ghana; UG, urban Ghana; EUR, Europe; PR, prevalence ratio. Research on Obesity and Diabetes among African Migrants (RODAM) Study. Ghana, United Kingdom, Germany, Netherlands 2012–2015.

The prevalence ratio of multimorbidity was higher in Europe (PR 1.47, 95% CI 1.35–1.58 in men, and 1.20, 1.12–1.27 in women) and urban Ghana (PR 1.45, 95% CI 1.30–1.58 in men, and 1.27, 1.20–1.34 in women) in a sex and age-adjusted analysis. Further adjustment for education and employment did not alter the results. Alcohol consumption and physical activity were initially included in the third model, but eventually they were excluded as the parsimonious model had a better fit based on Akaike Information Criterion (AIC). Adjustment for current smoking status (model three) did not change the rate of multimorbidity in Europe (PR 1.47, 95% CI 1.34–1.59 in men, and 1.18, 1.10–1.26 in women) and urban Ghana (PR 1.46, 95% CI 1.31–1.59 in men, and 1.27, 1.19–1.34 in women) compared to rural Ghana (Figure 4).

## Discussion

### Summary of Main Findings

The prevalence of individual chronic conditions varied per site and sex, and the prevalence of multimorbidity was higher for Ghanaian residents in urban areas (in Europe and Ghana) and lower in rural Ghana. In all study sites, multimorbidity was more prevalent in women than in men. A cardiometabolic pattern of multimorbidity was observed in all sites, while clustering of circulatory, metabolic and rheumatic disorders was observed only in Ghana. For both men and women, the rate of multimorbidity was higher for Ghanaian migrants in Europe and residents in urban Ghana compared to rural Ghana.

### Prevalence of NCDs and Multimorbidity: Comparison With Existing Literature

We observed a notably higher prevalence of rheumatoid disorders in Ghana compared to Ghanaian migrants in Europe. Previous research suggests that migrants in Europe have a higher burden of rheumatic disorders compared to the host population [31], however comparisons with the origin population are lacking. Although the possibility that these differences are caused by reporting bias cannot be completely ruled out, this is unlikely since the interviewers received specific training to avoid such issues. The multimorbidity prevalence estimates in our study, particularly in urban Ghana and Europe, are slightly higher than the findings from other studies in the general adult population in several HICs [10, 32–34] and LMICs [4, 5, 35]. The higher prevalence in the current study might be explained by different characteristics of the study population and/or inclusion of different NCDs in the definition of multimorbidity, including conditions such as obesity which is highly prevalent in the Ghanaian population. The higher prevalence of multimorbidity in women is a common finding [1, 4], and it is thought to result from biological differences or social factors such as a care-seeking behaviour and income inequalities [1]. Several characteristics are often presented as determinants of multimorbidity including older age, female sex and low socioeconomic status among others. However, these characteristics are already associated with an increased risk of individual chronic conditions, and their association with multimorbidity may be explained by the increased risk of accumulated individual conditions with a shared aetiology [1]. To guide prevention efforts more efficiently, further studies should investigate specific determinants of multimorbidity and of the most common disease clusters.

### Prevalence of Multimorbidity: Role of Migration and Urbanization

Ghanaian residents in Europe had a higher prevalence of multimorbidity compared to rural Ghana and similar to urban Ghana. Generally, other studies have observed a similar prevalence of multimorbidity in HICs compared to LMICs [6, 13]. Exposure to risk factors such as sedentary lifestyles, unhealthy diets and smoking are rapidly increasing in urban settings and could reflect the higher prevalence of multimorbidity observed in urban Ghanaian residents [6]. Migrants often represent a selected and healthier group of their population of origin. However, their health status may worsen over time [36], resulting with a higher prevalence of some NCDs and multimorbidity as compared to the host population [37]. The increased NCD and multimorbidity burden in migrants may arise from increased vulnerability to environmental, social, behavioural and psychosocial risks, including lifestyle changes, high-risk occupations, stress and discrimination [8]. Previous research in the RODAM Study has reported associations between duration of residence in Europe and acculturation and several risk factors [19, 38, 39]. This may partly explain the higher frequency of several non-communicable conditions in Europe compared to Ghana [17, 18] and of multimorbidity in Europe compared to rural Ghana.

Higher prevalence of cardiovascular risk factors and multimorbidity in urban areas has been observed in previous studies in Ghana and other LMICs [4, 6, 13]. Urbanization is thought to increase the risk of multimorbidity through environmental and lifestyle changes. The RODAM Study reports that the Ghanaian urban population has different dietary habits [40] and lower levels of physical activity compared to the rural Ghanaians (Table 1). Urbanization takes place at an unprecedented pace particularly in LMICs [41]. In this context, indications of increased risk of NCDs and multimorbidity in urban areas are alarming and prevention strategies need to be implemented.

### Patterns of Multimorbidity

Patterns of multimorbidity were shaped by the most prevalent diseases in the study population, in concordance with other studies [9]. Previous research also reported a cardiometabolic multimorbidity pattern consisting of non-communicable conditions like diabetes, obesity and hypertension [9, 13], and a highly frequent co-occurrence of cardiometabolic and rheumatic disorders in Ghana [9, 10, 13–15]. The differential patterns of multimorbidity that we observe in Europe and Ghana might be a consequence of changes in lifestyle, environmental exposures, healthcare access and socioeconomic position linked to migration and acculturation to the host country [39, 42]. While some multimorbidity patterns may share aetiological factors, such as diabetes and CVD being influenced by diet, the aetiology is unclear and probably heterogeneous for other combinations like rheumatic and cardiometabolic disorders. Knowledge on the factors driving differential patterns of disease may contribute to the design of efficient prevention and management strategies for such multimorbidity patterns. This suggests the need to explore the risk factors of different patterns of disease, particularly for the patterns with mixed aetiology in which prevention and management are not straightforward. The divergence in multimorbidity patterns between sites also indicates that multimorbidity is a complex phenomenon, and efforts must be made to understand the frequent combinations of disease, their occurrence and complications, while considering the setting and particularities of the population.

### Strengths and Limitations

The multi-centre study design of the RODAM Study includes areas in HICs where migrants live but also their populations of origin, which allows us to compare a relatively homogeneous population living in HICs and a middle-income country. This multi-centre design constitutes an important strength of the study as it provides a unique opportunity to study different settings and the effect of migration and urbanization on health. Furthermore, data collection procedures were highly standardized, ensuring comparability of the data from the five study sites.

The first limitation of the study refers to the different approaches to recruit participants in London and Berlin, which was based on Ghanaian association member lists and not on census due to different national regulations [37]. This difference in recruitment strategies, however, is unlikely to introduce bias as there were no substantial differences in the sociodemographic characteristics of the participants in locations using the census or the associations approach [17, 37, 20]. Non-respondent analysis showed that non-respondents were more often men (in all locations except for Berlin) and younger [17]. The analysis showed that although bias can never be completely ruled out in population studies, it was unlikely that the gender imbalance caused by non-response affected the NCDs prevalence estimates [17]. Another limitation of this study relates to the lack of information on some NCDs and communicable chronic conditions such as HIV or tuberculosis. The set-up of the RODAM Study allowed for the study of multimorbidity, however its cardiometabolic focus limited the range of diseases with available data. For some conditions, we had to rely on self-reported information obtained through a validated questionnaire, though generally assessment was based on laboratory measurements and medical diagnosis.

### Conclusion

The study of multimorbidity explores health outcomes in a comprehensive way, and this research shows that multimorbidity occurrence and patterns are affected by migration and urban living. Care for chronic conditions can benefit from a more holistic approach, transitioning from focusing on single diseases to an integrated model that considers multimorbidity and works on prevention and management beyond an index condition. Future studies should investigate factors driving differential risks of multimorbidity to assist prevention and management efforts, particularly in urban areas and rural regions undergoing urbanization processes such as in LMICs, as well as their migrant counterparts in HICs.

## Supplementary Material

Supplementary Figure S1 Flow-chart showing the number of individuals that agreed and participated in the study (6,385), with available data (5,659) and that were included in this analysis (4,833) in each study site (2,526 in Europe, 1,377 in rural Ghana, 990 in urban Ghana). The figure also shows the number of participants excluded because of lack of collected data (726) and because of missing data on multi-morbidity (826). Research on Obesity and Diabetes among African Migrants (RODAM). Ghana, United Kingdom, Germany, The Netherlands 2012–2015.

## References

[1] Academy of Medical Sciences. Multimorbidity: A Priority for Global Health Research. London: Academy of Medical Sciences (2018).

[2] Oni T McGrath N BeLue R Roderick P Colagiuri S May CR Chronic Diseases and Multi-Morbidity-Aa Conceptual Modification to the WHO ICCC Model for Countries in Health Transition. BMC Public Health (2014) 14(575):575–7. 10.1186/1471-2458-14-575 24912531 PMC4071801

[3] Abbafati C Abbas KM Abbasi-Kangevari M Abd-Allah F Abdelalim A Abdollahi M Global burden of 369 Diseases and Injuries in 204 Countries and Territories, 1990-2019: a Systematic Analysis for the Global Burden of Disease Study 2019. Lancet (2020) 396(10258):1204–22. 10.1016/S0140-6736(20)30925-9 33069326 PMC7567026

[4] Arokiasamy P Uttamacharya U Jain K Biritwum RB Yawson AE Wu F The Impact of Multimorbidity on Adult Physical and Mental Health in Low- and Middle-Income Countries: What Does the Study on Global Ageing and Adult Health (SAGE) Reveal? BMC Med (2015) 13(1):178–16. 10.1186/s12916-015-0402-8 26239481 PMC4524360

[5] Abebe F Schneider M Asrat B Ambaw F. Multimorbidity of Chronic Non-communicable Diseases in Low- and Middle-Income Countries: A Scoping Review. J Comorb (2020) 10:2235042X2096191. 10.1177/2235042x20961919 PMC7573723 33117722

[6] Afshar S Roderick PJ Kowal P Dimitrov BD Hill AG. Multimorbidity and the Inequalities of Global Ageing: A Cross-Sectional Study of 28 Countries Using the World Health Surveys. BMC Public Health (2015) 15(1):776–10. 10.1186/s12889-015-2008-7 26268536 PMC4534141

[7] Gimeno-Feliu LA Calderón-Larrañaga A Díaz E Laguna-Berna C Poblador-Plou B Coscollar C Multimorbidity and Immigrant Status: Associations with Area of Origin and Length of Residence in Host Country. Fam Pract (2017) 34(6):662–6. 10.1093/fampra/cmx048 29106530

[8] Diaz E Kumar BN Gimeno‐Feliu LA Calderón‐Larrañaga A Poblador‐Pou B Prados‐Torres A. Multimorbidity Among Registered Immigrants in Norway: The Role of Reason for Migration and Length of Stay. Trop Med Int Health (2015) 20(12):1805–14. 10.1111/tmi.12615 26426974

[9] Violan C Foguet-Boreu Q Flores-Mateo G Salisbury C Blom J Freitag M Prevalence, Determinants and Patterns of Multimorbidity in Primary Care: A Systematic Review of Observational Studies. PLoS One (2014) 9(7):e102149–11. 10.1371/journal.pone.0102149 25048354 PMC4105594

[10] Agborsangaya CB Lau D Lahtinen M Cooke T Johnson JA. Multimorbidity Prevalence and Patterns across Socioeconomic Determinants: a Cross-Sectional Survey. BMC Public Health (2012) 12(1):201. 10.1186/1471-2458-12-201 22429338 PMC3353224

[11] Roth GA Abate D Abate KH Abay SM Abbafati C Abbasi N Global, Regional, and National Age-sex-specific Mortality for 282 Causes of Death in 195 Countries and Territories, 1980-2017: a Systematic Analysis for the Global Burden of Disease Study 2017. Lancet (2018) 392(10159):1736–88. 10.1016/S0140-6736(18)32203-7 30496103 PMC6227606

[12] Agrawal G Patel SK Agarwal AK. Lifestyle Health Risk Factors and Multiple Non-communicable Diseases Among the Adult Population in India: a Cross-Sectional Study. J Public Health (2016) 24(4):317–24. 10.1007/s10389-016-0727-6

[13] Garin N Koyanagi A Chatterji S Tyrovolas S Olaya B Leonardi M Global Multimorbidity Patterns: A Cross-Sectional, Population-Based, Multi-Country Study. Gerona (2016) 71(2):205–14. 10.1093/gerona/glv128 PMC5864156 26419978

[14] Hien H Berthé A Drabo MK Meda N Konaté B Tou F Prevalence and Patterns of Multimorbidity Among the Elderly in Burkina Faso: Cross-Sectional Study. Trop Med Int Health (2014) 19(11):1328–33. 10.1111/tmi.12377 25164626

[15] Morgan SA Eyles C Roderick PJ Adongo PB Hill AG. Women Living with Multi-Morbidity in the Greater Accra Region of Ghana: a Qualitative Study Guided by the Cumulative Complexity Model. J Biosoc Sci (2018) 1–16. 10.1017/s0021932018000342 30472965

[16] Grabovschi C Loignon C Fortin M. Mapping the Concept of Vulnerability Related to Health Care Disparities: A Scoping Review. BMC Health Serv Res (2013) 13(1):94. 10.1186/1472-6963-13-94 23496838 PMC3626765

[17] Agyemang C Meeks K Beune E Owusu-Dabo E Mockenhaupt FP Addo J Obesity and Type 2 Diabetes in Sub-saharan Africans – Is the burden in Today’s Africa Similar to African Migrants in Europe? the RODAM Study Internal and Emergency Medicine (2016). 10.1186/s12916-016-0709-0 PMC5075171 27769239

[18] Agyemang C Nyaaba G Beune E Meeks K Owusu-Dabo E Addo J Variations in Hypertension Awareness, Treatment, and Control Among Ghanaian Migrants Living in Amsterdam, Berlin, London, and Nonmigrant Ghanaians Living in Rural and Urban Ghana - the RODAM Study. J Hypertens (2018) 36(1):169–77. 10.1097/hjh.0000000000001520 28858173

[19] Boateng D Agyemang C Beune E Meeks K Smeeth L Schulze M Migration and Cardiovascular Disease Risk Among Ghanaian Populations in Europe: The RODAM Study (Research on Obesity and Diabetes Among African Migrants). Circ Cardiovasc Qual Outcomes (2017) 10(11):e004013. 10.1161/CIRCOUTCOMES.117.004013 29150534

[20] Agyemang C Beune E Meeks K Owusu-Dabo E Agyei-Baffour P Aikins A Rationale and Cross-Sectional Study Design of the Research on Obesity and Type 2 Diabetes Among African Migrants: The RODAM Study. BMJ Open (2014) 4(3):e004877–9. 10.1136/bmjopen-2014-004877 PMC3963103 24657884

[21] International IPAQ. Physical Activity Questionnaire. Guidelines for Data Processing and Analysis of the International Physical Activity Questionnaire. IPAQ) (2005).

[22] Catapano AL Graham I De Backer G Wiklund O Chapman MJ Drexel H 2016 ESC/EAS Guidelines for the Management of Dyslipidaemias. Eur Heart J (20162016) 37:2999–3058. 10.1093/eurheartj/ehw272 27567407

[23] World Health Organization, International Diabetes Federation. Definition and Diagnosis of Diabetes Mellitus and Intermediate Hyperglycemia: Report of a WHO/IDF Consultation (2006).

[24] KDIGO Work Group. KDIGO Clinical Practice Guideline for Glomerulo- Nephritis. Kidney Int Suppl (2012) 2(2):139–274.

[25] Adjei DN Stronks K Adu D Beune E Meeks K Smeeth L Chronic Kidney Disease burden Among African Migrants in Three European Countries and in Urban and Rural Ghana: the RODAM Cross-Sectional Study. Nephrol Dial Transpl (2018) 33(10):1812–22. 10.1093/ndt/gfx347 29342308

[26] Rose GA. The Diagnosis of Ischaemic Heart Pain and Intermittent Claudication in Field Surveys. Bull World Health Organ (1962) 27:645–58. 13974778 PMC2555832

[27] Kroenke K Spitzer RL Williams JBW Löwe B. The Patient Health Questionnaire Somatic, Anxiety, and Depressive Symptom Scales: A Systematic Review. Gen Hosp Psychiatry (2010) 32(4):345–59. 10.1016/j.genhosppsych.2010.03.006 20633738

[28] World Health Organization (Who). ICD-11 for Mortality and Morbidity Statistics (2019).

[29] Anderson RN Rosenberg HM. Age Standardization of Death Rates: Implementation of the Year 2000 Standard. Natl Vital Stat Rep (1998) 47(3):1–20. 9796247

[30] RStudio Team. RStudio. Boston, MA: Integrated Development Environment for RRStudio, Inc. (2016).

[31] Bernd R Mladovsky P Deville W Rijks B Petrova-Benedict R McKee M. Migration and Health in the European Union, Tropical Medicine and International Health, Vol. 3. Maidenhead, New York: Open University Press (2011). p. 109.

[32] Marengoni A Angleman S Melis R Mangialasche F Karp A Garmen A Aging with Multimorbidity: A Systematic Review of the Literature. Ageing Res Rev (2011) 10(4):430–9. 10.1016/j.arr.2011.03.003 21402176

[33] Barnett K Mercer SW Norbury M Watt G Wyke S Guthrie B. Epidemiology of Multimorbidity and Implications for Health Care, Research, and Medical Education: A Cross-Sectional Study. The Lancet (2012) 380(9836):37–43. 10.1016/s0140-6736(12)60240-2 22579043

[34] Puth MT Weckbecker K Schmid M Münster E. Prevalence of Multimorbidity in Germany: Impact of Age and Educational Level in a Cross-Sectional Study on 19,294 Adults. BMC Public Health (2017) 17(1):826–7. 10.1186/s12889-017-4833-3 29047341 PMC5648462

[35] Pati S Swain S Knottnerus JA Metsemakers JFM Van Den Akker M. Health Related Quality of Life in Multimorbidity: A Primary-Care Based Study from Odisha, India. Health Qual Life Outcomes (2019) 17(1):116–1. 10.1186/s12955-019-1180-3 31277648 PMC6612103

[36] Diaz E Poblador-Pou B Gimeno-Feliu LA Calderón-Larrañaga A Kumar BN Prados-Torres A. Multimorbidity and its Patterns According to Immigrant Origin. A Nationwide Register-Based Study in Norway. PLoS One (2015) 10(12):e0145233–18. 10.1371/journal.pone.0145233 26684188 PMC4684298

[37] Agyemang C Beune E Meeks K Addo J Aikins AD Bahendeka S Innovative Ways of Studying the Effect of Migration on Obesity and Diabetes beyond the Common Designs: Lessons from the RODAM Study. Ann N Y Acad Sci (2017) 1391(1):54–70. 10.1111/nyas.13204 27706830

[38] Addo J Cook S Galbete C Agyemang C Klipstein-Grobusch K Nicolaou M Differences in Alcohol Consumption and Drinking Patterns in Ghanaians in Europe and Africa: The RODAM Study. PLoS One (2018) 13(11):e0206286. 10.1371/journal.pone.0206286 30388130 PMC6214514

[39] Brathwaite R Addo J Kunst AE Agyemang C Owusu-Dabo E De-Graft Aikins A Smoking Prevalence Differs by Location of Residence Among Ghanaians in Africa and Europe: The RODAM Study. PLoS One (2017) 12(5):e0177291–15. 10.1371/journal.pone.0177291 28475620 PMC5419606

[40] Galbete C Nicolaou M Meeks KA de-Graft Aikins A Addo J Amoah SK Food Consumption, Nutrient Intake, and Dietary Patterns in Ghanaian Migrants in Europe and Their Compatriots in Ghana. Food Nutr Res (2017) 61(1):1341809. 10.1080/16546628.2017.1341809 28747862 PMC5510194

[41] Aliyu A Amadu L. Urbanization, Cities, and Health: The Challenges to Nigeria - A Review. Ann Afr Med (2017) 16(4):149–58. 10.4103/aam.aam_1_17 29063897 PMC5676403

[42] Chilunga FP Boateng D Henneman P Beune E Requena-Méndez A Meeks K Perceived Discrimination and Stressful Life Events Are Associated with Cardiovascular Risk Score in Migrant and Non-migrant Populations: The RODAM Study. Int J Cardiol (2019) 286:169–74. 10.1016/j.ijcard.2018.12.056 30638750

