# Supplementary material for: Multimorbidity Among Migrant and Non-Migrant Ghanaians: The RODAM Study
Source: Int J Public Health. 2021 Dec 31;66:1604056. doi: 10.3389/ijph.2021.1604056 (PMC8759292; doi:10.3389/ijph.2021.1604056)
Supplement: Supplementary file 1 [file Table1.docx]

**Supplementary Table 1:** Criteria to define study population characteristics and non-communicable diseases included in the study.

| **Health condition** | **Definition** | **Data collection procedure** | **Guideline or study of reference** |
| --- | --- | --- | --- |
| Hypertension | Blood pressure was measured three times in a sitting position after at least five minutes of rest. Hypertension was defined as systolic BP≥140mm Hg, or diastolic BP≥90mm Hg, or being on antihypertensive medication. | RODAM Questionnaire, physical examination. | Agyemang C, Nyaaba G, Beune E, et al. Variations in hypertension awareness, treatment, and control among Ghanaian migrants living in Amsterdam, Berlin, London, and nonmigrant Ghanaians living in rural and urban Ghana-the RODAM study. J. Hypertens. 36(1) 2018.  Mancia G, Fagard R, Narkiewicz K, et al. 2013 ESH/ESC guidelines for the management of arterial hypertension: The Task Force for the management of arterial hypertension of the European Society of Hypertension (ESH) and of the European Society of Cardiology (ESC). Eur. Heart J. 34(28) 2013. |
| Obesity | Weight and height were measured in light clothing without shoes using SECA 877 weighing scales and SECA 217 portable stadiometers. BMI (kg/m2) was calculated by dividing the weight in kilograms by the squared height in meters. Obesity was defined as BMI ≥30 kg/m^2^ | Physical examination |  |
| Type 2 diabetes mellitus | Type 2 diabetes mellitus was as fasting plasma glucose ≥7.0mmol/L, or current use of medication prescribed for diabetes, or self-reported diabetes | RODAM Questionnaire, laboratory measurements | World Health Organization. Definition and diagnosis of diabetes mellitus and intermediate hyperglycemia: report of a WHO/IDF consultation. 2006 . |
| Hypercholesterolemia | Hypercholesterolemia was defined as total cholesterol level ≥5mmol/L. | Laboratory measurements | van der Linden E, Meeks K, Beune E, et al. Dyslipidaemia among Ghanaian migrants in three European countries and their compatriots in rural and urban Ghana: The RODAM study. Atherosclerosis 284 2019.  Perk J, De Backer G, Gohlke H,et al. European Guidelines on cardiovascular disease prevention in clinical practice (version 2012). Eur. Heart J. 33(13) 2012. |
| Cardiovascular disease | Prevalent CVD (intermittent claudication, angina pectoris and possible myocardial infarction) | RODAM Questionnaire | Rose GA. The diagnosis of ischaemic heart pain and intermittent claudication in field surveys. Bull World Health Organ. 1962;27:645–58. |
| Chronic Kidney disease | Estimated glomerular filtration rate and albuminuria were used to estimate severity of chronic kidney disease according to the 2012 KDIGO classification KDIGO Work Group. KDIGO clinical practice guideline for glomerulo- nephritis. Kidney Int. Suppl. 2(2) 2012.; . : low, moderately increased, high and very high risk. Participants with moderately increased, high and very high risk of chronic kidney disease were considered to have kidney disease. | Laboratory measurements | KDIGO Work Group. KDIGO clinical practice guideline for glomerulo- nephritis. Kidney Int. Suppl. 2(2) 2012. |
| Rheumatic disorders | Inflammatory rheumatism, chronic rheumatism or rheumatoid arthritis diagnosed by a doctor | RODAM Questionnaire |  |
| Depressive symptoms | Score of >=10 in the Patient Health Questionnaire (PHQ)-9. The questionnaires includes the following items: in the last 2 weeks, have you felt: little interest or pleasure in doing things; feeling down, depressed or hopeless; trouble falling or staying asleep, or sleeping too much; feeling tired; poor appetite or overeating, feeling bad about yourself or that you are a failure; trouble concentrating; moving or speaking so slowly that other people could have noticed, or being more fidgety or restless than usual; thoughts that you would be better off dead, or of hurting yourself; feeling stressed due to financial demands from relatives or friends in Ghana; feeling homesick; feelings stressed due to demands of host country (rules, fast way of living). | RODAM Questionnaire | Kroenke K, Spitzer RL, Williams JBW, et al. The Patient Health Questionnaire Somatic, Anxiety, and Depressive Symptom Scales: A systematic review. Gen. Hosp. Psychiatry 32(4) 2010. |
