# Supplementary figures and images for: Multimorbidity Among Migrant and Non-Migrant Ghanaians: The RODAM Study
Source: Int J Public Health. 2021 Dec 31;66:1604056. doi: 10.3389/ijph.2021.1604056 (PMC8759292; doi:10.3389/ijph.2021.1604056)

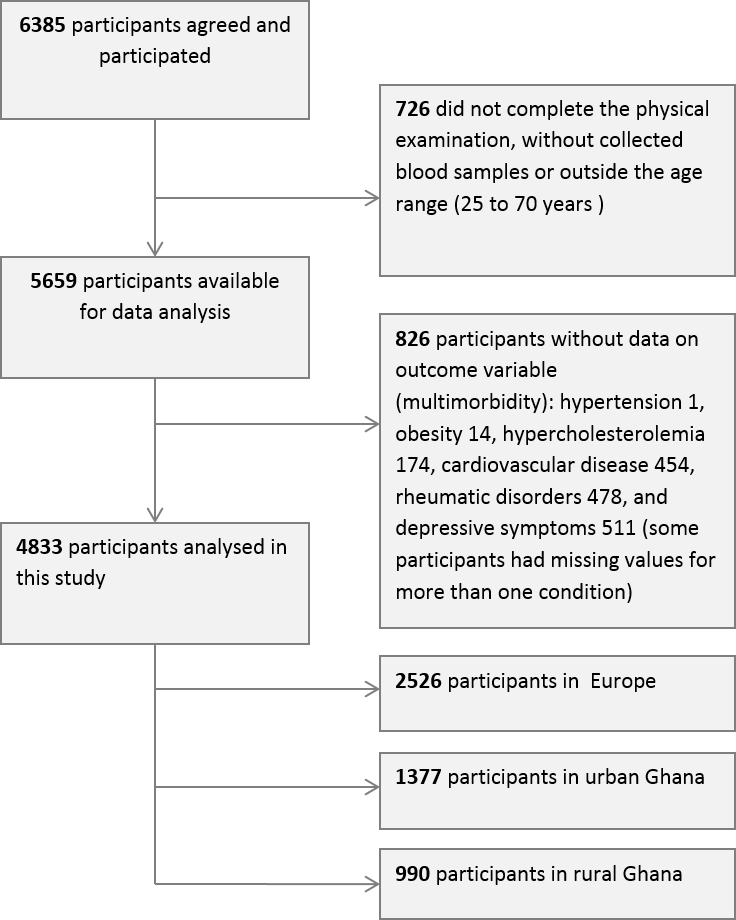

Supplement: Supplementary file 2 [file Image1.TIF]
