# Supplementary material for: Multimorbidity Among Migrant and Non-Migrant Ghanaians: The RODAM Study
Source: Int J Public Health. 2021 Dec 31;66:1604056. doi: 10.3389/ijph.2021.1604056 (PMC8759292; doi:10.3389/ijph.2021.1604056)
Supplement: Supplementary file 3 [file Table2.docx]

**Supplementary Table 2**: Prevalence of chronic conditions stratified by sex, site and age groups.

| **Men** | **Site** |  | **Europe** | | |  | **Urban Ghana** | | |  | **Rural Ghana** | | |  | **Total** | | |
| --- | --- | --- | --- | --- | --- | --- | --- | --- | --- | --- | --- | --- | --- | --- | --- | --- | --- |
|  | **Age** |  | **25-41** | **42-53** | **54-70** |  | **25-41** | **42-53** | **54-70** |  | **25-41** | **42-53** | **54-70** |  | **25-41** | **42-53** | **54-70** |
|  |  |  | **(N=307)** | **(N=442)** | **(N=326)** |  | **(N=135)** | **(N=130)** | **(N=121)** |  | **(N=148)** | **(N=100)** | **(N=119)** |  | **(N=590)** | **(N=672)** | **(N=566)** |
| **Hypertension** | | | 88 (28.7) | 261 (59.0) | 250 (76.7) |  | 19 (14.1) | 41 (31.5) | 73 (60.3) |  | 10 (6.8) | 26 (26.0) | 47 (39.5) |  | 117 (19.8) | 328 (48.8) | 370 (65.4) |
| **Obesity** | | | 41 (13.4) | 78 (17.6) | 71 (21.8) |  | 7 (5.2) | 13 (10.0) | 5 (4.1) |  | 2 (1.4) | 1 (1.0) | 0 (0) |  | 50 (8.5) | 92 (13.7) | 76 (13.4) |
| **Diabetes Mellitus** | | | 16 (5.2) | 60 (13.6) | 71 (21.8) |  | 4 (3.0) | 9 (6.9) | 32 (26.4) |  | 1 (0.7) | 3 (3.0) | 9 (7.6) |  | 21 (3.6) | 72 (10.7) | 112 (19.8) |
| **Hypercholesterolemia** | | | 126 (41.0) | 224 (50.7) | 175 (53.7) |  | 53 (39.3) | 77 (59.2) | 65 (53.7) |  | 29 (19.6) | 22 (22.0) | 25 (21.0) |  | 208 (35.3) | 323 (48.1) | 265 (46.8) |
| **Cardiovascular Disease** | | | 18 (5.9) | 33 (7.5) | 36 (11.0) |  | 28 (20.7) | 19 (14.6) | 21 (17.4) |  | 38 (25.7) | 25 (25.0) | 20 (16.8) |  | 84 (14.2) | 77 (11.5) | 77 (13.6) |
| **Chronic Kidney Disease** | | | 21 (6.8) | 38 (8.6) | 42 (12.9) |  | 9 (6.7) | 13 (10.0) | 35 (28.9) |  | 6 (4.1) | 5 (5.0) | 11 (9.2) |  | 36 (6.1) | 56 (8.3) | 88 (15.5) |
| **Rheumatic Disorders** | | | 10 (3.3) | 30 (6.8) | 23 (7.1) |  | 44 (32.6) | 57 (43.8) | 34 (28.1) |  | 44 (29.7) | 33 (33.0) | 43 (36.1) |  | 98 (16.6) | 120 (17.9) | 100 (17.7) |
| **Depressive symptoms** | | | 19 (6.2) | 25 (5.7) | 20 (6.1) |  | 6 (4.4) | 3 (2.3) | 1 (0.8) |  | 4 (2.7) | 10 (10.0) | 4 (3.4) |  | 29 (4.9) | 38 (5.7) | 25 (4.4) |
| **Multimorbidity** | | | 97 (31.6) | 239 (54.1) | 228 (69.9) |  | 43 (31.9) | 71 (54.6) | 81 (66.9) |  | 35 (23.6) | 32 (32.0) | 46 (38.7) |  | 175 (29.7) | 342 (50.9) | 355 (62.7) |
| **Women** | **Site** |  | **Europe** | | |  | **Urban Ghana** | | |  | **Rural Ghana** | | |  | **Total** | | |
|  | **Age** |  | **25-41** | **42-53** | **54-70** |  | **25-41** | **42-53** | **54-70** |  | **25-41** | **42-53** | **54-70** |  | **25-41** | **42-53** | **54-70** |
|  |  |  | **(N=479)** | **(N=659)** | **(N=313)** |  | **(N=404)** | **(N=345)** | **(N=242)** |  | **(N=204)** | **(N=187)** | **(N=172)** |  | **(N=1087)** | **(N=1191)** | **(N=727)** |
| **Hypertension** | | | 116 (24.2) | 368 (55.8) | 234 (74.8) |  | 46 (11.4) | 124 (35.9) | 120 (49.6) |  | 23 (11.3) | 58 (31.0) | 75 (43.6) |  | 185 (17.0) | 550 (46.2) | 429 (59.0) |
| **Obesity** | | | 194 (40.5) | 343 (52.0) | 169 (54.0) |  | 121 (30.0) | 125 (36.2) | 88 (36.4) |  | 20 (9.8) | 14 (7.5) | 11 (6.4) |  | 335 (30.8) | 482 (40.5) | 268 (36.9) |
| **Diabetes Mellitus** | | | 13 (2.7) | 59 (9.0) | 63 (20.1) |  | 14 (3.5) | 38 (11.0) | 33 (13.6) |  | 5 (2.5) | 10 (5.3) | 20 (11.6) |  | 32 (2.9) | 107 (9.0) | 116 (16.0) |
| **Hypercholesterolemia** | | | 159 (33.2) | 334 (50.7) | 189 (60.4) |  | 185 (45.8) | 218 (63.2) | 180 (74.4) |  | 38 (18.6) | 80 (42.8) | 89 (51.7) |  | 382 (35.1) | 632 (53.1) | 458 (63.0) |
| **Cardiovascular Disease** | | | 35 (7.3) | 69 (10.5) | 36 (11.5) |  | 69 (17.1) | 65 (18.8) | 58 (24.0) |  | 48 (23.5) | 38 (20.3) | 53 (30.8) |  | 152 (14.0) | 172 (14.4) | 147 (20.2) |
| **Chronic Kidney Disease** | | | 45 (9.4) | 78 (11.8) | 34 (10.9) |  | 31 (7.7) | 53 (15.4) | 48 (19.8) |  | 13 (6.4) | 19 (10.2) | 42 (24.4) |  | 89 (8.2) | 150 (12.6) | 124 (17.1) |
| **Rheumatic Disorders** | | | 16 (3.3) | 34 (5.2) | 18 (5.8) |  | 143 (35.4) | 135 (39.1) | 113 (46.7) |  | 66 (32.4) | 64 (34.2) | 83 (48.3) |  | 225 (20.7) | 233 (19.6) | 214 (29.4) |
| **Depressive symptoms** | | | 39 (8.1) | 58 (8.8) | 24 (7.7) |  | 13 (3.2) | 15 (4.3) | 14 (5.8) |  | 8 (3.9) | 17 (9.1) | 19 (11.0) |  | 60 (5.5) | 90 (7.6) | 57 (7.8) |
| **Multimorbidity** | | | 179 (37.4) | 437 (66.3) | 258 (82.4) |  | 188 (46.5) | 239 (69.3) | 195 (80.6) |  | 62 (30.4) | 96 (51.3) | 122 (70.9) |  | 429 (39.5) | 772 (64.8) | 575 (79.1) |
